# PIWIL1 suppresses circadian rhythms through GSK3β‐induced phosphorylation and degradation of CLOCK and BMAL1 in cancer cells

**DOI:** 10.1111/jcmm.14377

**Published:** 2019-05-16

**Authors:** Hao Tan, Yingchuan Zhu, Xulei Zheng, Yilu Lu, Dachang Tao, Yunqiang Liu, Yongxin Ma

**Affiliations:** ^1^ Department of Medical Genetics, State Key Laboratory of Biotherapy West China Hospital, Sichuan University and Collaborative Innovation Center Chengdu China

**Keywords:** circadian rhythms, degradation, GSK3β, phosphorylation, PIWIL1, ubiquitination

## Abstract

Circadian rhythms are maintained by series of circadian clock proteins, and post‐translation modifications of clock proteins significantly contribute to regulating circadian clock. However, the underlying upstream mechanism of circadian genes that are responsible for circadian rhythms in cancer cells remains unknown. PIWIL1 participates in many physiological processes and current discoveries have shown that PIWIL1 is involved in tumorigenesis in various cancers. Here we report that PIWIL1 can suppress circadian rhythms in cancer cells. Mechanistically, by promoting SRC interacting with PI3K, PIWIL1 can activate PI3K‐AKT signalling pathway to phosphorylate and inactivate GSK3β, repressing GSK3β‐induced phosphorylation and ubiquitination of CLOCK and BMAL1. Simultaneously, together with CLOCK/BMAL1 complex, PIWIL1 can bind with E‐BOX region to suppress transcriptional activities of clock‐controlled genes promoters. Collectively, our findings first demonstrate that PIWIL1 negatively regulates circadian rhythms via two pathways, providing molecular connection between dysfunction of circadian rhythms and tumorigenesis.

## 1 INTRODUCTION

Circadian rhythms are existed in almost all species and show 24 hours’ cycles. In mammals, circadian clock is maintained by a master clock in hypothalamus suprachiasmatic nucleus, and clocks are also located in most other tissues. The regulation of circadian clock depends on self‐continued transcription‐translation feedback loops.^1^, ^2^, ^3^, ^4^ In mammals, the core clock genes are *CLOCK* and *BMAL1*. CLOCK (Circadian locomotor output cycles kaput) dimerizes with BMAL1 (Brain‐Muscle Arnt‐Like protein 1) to activate circadian output genes via binding to their E‐BOX region on promoter, for example, Period (PER) 1/2, Cryptochrome (CRY) 1/2. Then PER1/2 and CRY1/2 enter into the nucleus to suppress activity of CLOCK/BMAL1, thereby inhibiting their own transcription.^2^ And the other feedback loops are generated by the *Rev‐erb* and *Ror* genes, whose proteins regulate the *BMAL1* gene transcription.^5^, ^6^, ^7^

Previous studies have shown that the clock proteins are subject to phosphorylation, ubiquitination or other post‐translational modifications (PTMS).^8^ Recently, GSK3β (glycogen synthase kinase 3) has been confirmed as a crucial regulator for stability and activity of clock proteins, including CLOCK, BMAL1, PER and CRY.^9^, ^10^, ^11^, ^12^ In general, GSK3β can phosphorylate clock proteins, thus leading to ubiquitination and proteasome‐dependent degradation. However, the upstream mechanism controlling the circadian system has not been better understood.

In human, PIWI protein subfamily mainly includes PIWIL1, PIWIL2, PIWIL3 and PIWIL4, and participates in stem cell self‐renewal, spermatogenesis and RNA silencing.^13^, ^14^, ^15^ PIWIL1 (Piwi‐like RNA‐mediated gene silencing 1), also known as HIWI, contains two conserved domains: the PAZ domain in middle and the PIWI domain in C‐terminal.^16^ Current discoveries have shown that PIWIL1 is involved in tumorigenesis in various cancers, including proliferation, migration and invasion of cancers.^17^, ^18^, ^19^, ^20^ Meanwhile, evidences have demonstrated that the dysfunction of circadian rhythms is associated with tumours.^21^, ^22^ Yet, the link between PIWIL1 and circadian rhythms remains unclear, and then we have proposed the hypothesis that PIWIL1 can regulate circadian rhythms.

Here we report that PIWIL1 can suppress circadian rhythms in cancer cells. The results revealed that PIWIL1 can activate SRC‐PI3K‐AKT signalling pathway, thus leading to phosphorylation of GSK3β, repressing GSK3β‐induced phosphorylation and degradation of CLOCK and BMAL1. Simultaneously, together with CLOCK and BMAL1 complex, PIWIL1 can bind with E‐BOX region to inhibit the transcriptional activities of clock‐controlled genes (CCG) promoters. Our discoveries provide a novel molecular connection between dysfunction of circadian rhythms and tumorigenesis.

## 2 MATERIALS AND METHODS

### 2.1 Plasmid construction and shRNA

cDNA encoding MYC‐tagged PIWIL1 and MYC‐tagged PIWIL1 deletion mutants were synthesized and inserted into pcDNA3.1(+) expression vector (Invitrogen) by segment and fusion PCR. Meanwhile, PIWIL1‐specific shRNA (shPIWIL1) was synthesized and cloned into pGPU6/GFP/Neo (GenePharma, Shanghai, China). The target sequence of shPIWIL1 was 5'‐GCCGTTCATACAAGACTAATT‐3'.

### 2.2 Antibody

The whole rabbit polyclonal antibodies (pAbs) and mouse monoclonal antibodies (mAbs) were listed below: pAb anti‐PIWIL1 (Abcam, Cambridge, UK), pAb anti‐CLOCK (CST, Boston), pAb anti‐BMAL1 (CST), pAb anti‐MYC‐tag (Santa Cruz, USA), pAb anti‐HA‐tag (Santa), mAb anti‐GAPDH (Abcam), mAb anti‐GSK3β (Zen Bioscience, Chengdu, China), pAb anti‐pGSK3β(S9) (CST), pAb anti‐AKT (CST), pAb anti‐pAKT(S473) (CST), pAb anti‐SRC (CST), pAb anti‐PER2 (CST), pAb anti‐CRY1 (CST).

### 2.3 Cell culture and treatment

293T, Hela and MCF7 cells were maintained in State Key Laboratory of Biotherapy of West China Hospital. The cells were cultured in DMEM containing 10% FBS and transfected with jetPRIME (#114‐15, SA, France), then incubated in 5% CO2 incubator at 37°C. For treatment, after transfection, cells were pre‐treated with 6 μg/mL cycloheximide (CHX) for 0‐8 hours to inhibit protein synthesis, 10 μmol/L MG132 for 6 hours to suppress protein degradation, 10 μmol/L TWS119 for 6 hours (inhibitor of GSK3β), 50 μmol/L LY294002 (inhibitor of PI3K) for 4 hours, 1 μmol/L Saracatinib and Bosutinib (AZD, SKI, inhibitors of SRC) for 4 hours, respectively. Then the cells were harvested and analysed using appropriate antibodies. All experiments were repeated at three times.

### 2.4 Immunoprecipitation and Western blot

For immunoprecipitation (IP), cells were lysed by using protein extraction buffers (PP1801, Bioteke, China) containing protease inhibitor (04693116001, Roche, Switzerland) after transfected for 48 hours. The proteins extracted were immunoprecipitated with specific antibody and protein A + G agarose beads (P2012, Beyotime, China). For Western blot, proteins were separated with SDS‐PAGE and detected with special primary antibody and horseradish peroxidase‐conjugated secondary antibodies. Then special proteins were visualized with the enhanced chemiluminescence Western blot detection system (WBKLS0100, Millipore).

### 2.5 Immunofluorescence

The cells were fixed using 4% paraformaldehyde in PBS for 10 minutes and permeabilized for 3 minutes with 0.5% Triton, and blocked for 30 minutes with 1% BSA, incubated with primary antibody for overnight at 4°C, and next incubated with Alexa Fluor^®^ 488 (A‐11055; Thermo Fisher Scientific, USA), Alexa Fluor^®^ 555 (A‐31570; Thermo Fisher Scientific, USA) and Alexa Fluor^®^ 647 (132570; Jackson Immuno Research, USA) at room temperature for 1 hour, each step was followed with two washes for 5 minutes in PBS. Finally, the specimens were counterstained with DAPI (1‰) for 2 minutes nuclear stain. The images were obtained using confocal microscope (Olympus, Japan).

### 2.6 Chromatin immunoprecipitation and quantitative PCR

Chromatin immunoprecipitation (ChIP) was performed with the ChIP Assay Kit (Beyotime, China) according to manufacturer's protocols. Cells were fixed with formaldehyde for 10 minutes, crosslinking was stopped with 125 mmol/L glycine, and lysed using SDS lysis buffer with protease inhibitor cocktail (Roche). Cell lysates were ultrasonicated and immunoprecipitated with antibody. The DNA eluted and purified was subjected to qPCR with primers as below: Per2 sense: 5'‐GGACGACGGGTAGCACGAA‐3'; Per2 antisense: 5'‐GCCGCTGTCACATAGTGGAAAA‐3'; CRY1 sense: 5'‐CATAGAGGCAGGAAGGAGAA‐3'; CRY1 antisense: 5'‐ATCAGCCTTTCTTTGGTTCT‐3'. For quantitative PCR, it was performed by real‐time PCR system (BioRad, Hercules, CA), with the first denaturation step at 95°C for 10 minutes, followed with 40 cycles containing denaturation at 95°C for 20 seconds, annealing at 60°C for 30 seconds and extension at 72°C for 30 seconds.

### 2.7 Dual luciferase activity assay

For dual luciferase activity assay, E‐BOX sequences of *PER2* and *CRY1* were inserted into pGL3 reporter vectors (Promega, WI). A total of 0.45 μg plasmid and 0.1 μg renilla plasmid were co‐transfected into Hela cells. After 48 hours, cells were harvested using Luciferase Assay Buffer, then detected with Luciferase Assay Reagent II and Stop & Glo Reagent according to the manufacturer's protocols.

### 2.8 Statistical analysis

The whole experiments were repeated for three times, and the data were presented as the mean ± SD with GraphPad Prism 7.0 software. The statistical analyses were analysed with spss version 17.0. Differences were determined with Student's *t* test between the experimental groups. Statistical significance were considered as *P* < 0.05.

## 3 RESULTS

### 3.1 PIWIL1 increases CLOCK and BMAL1 expression

**Figure 1:**
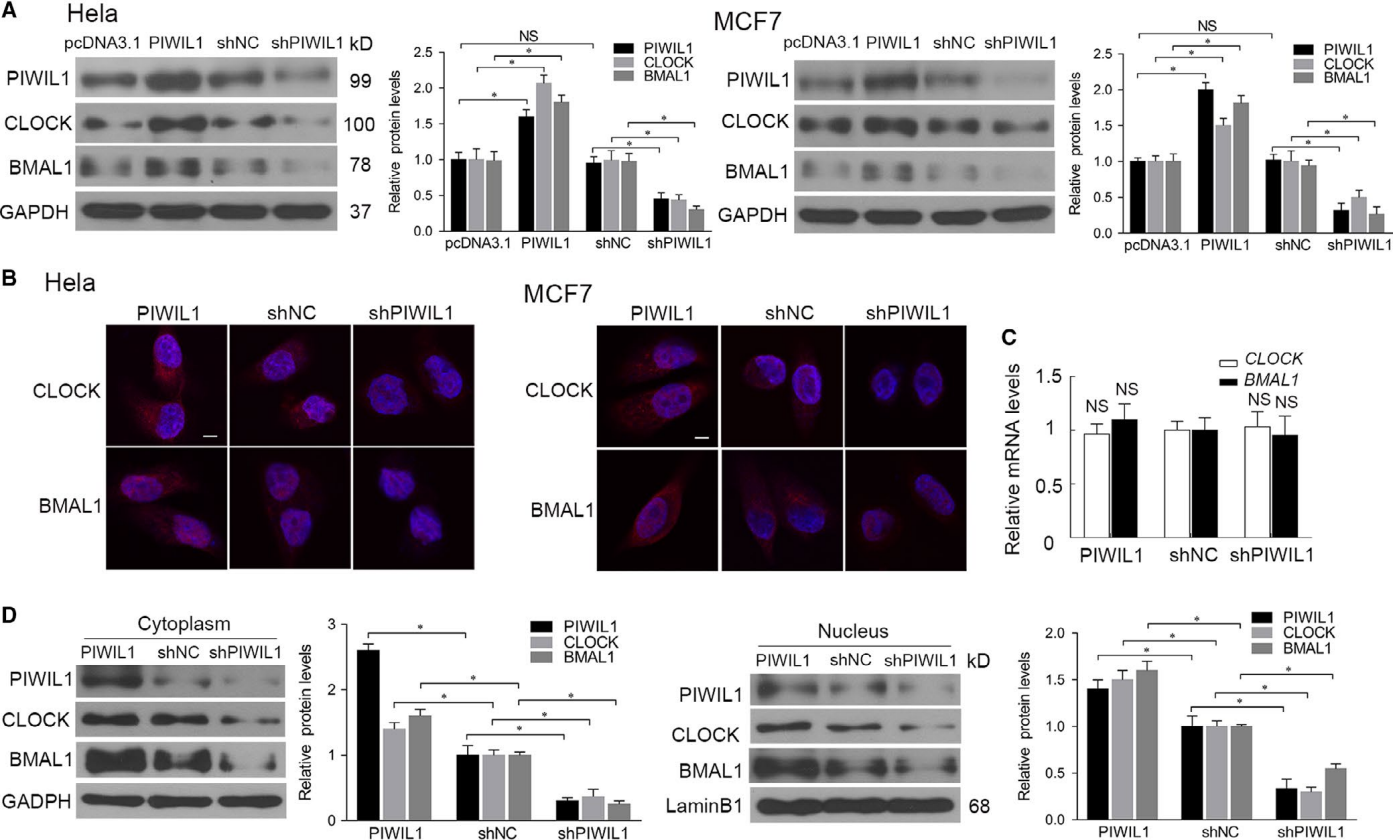
PIWIL1 increases CLOCK and BMAL1 expression. PIWIL1 positively regulated CLOCK and BMAL1 expression in Hela and MCF7 cells compared with Control transfected with empty vector (pcDNA3.1) and shRNA vector (shNC) (A). Fluorescent staining of CLOCK and BMAL1 decreased in PIWIL1 knockdown cells, Scale bars correspond to 5 μm (B). qPCR results showed that PIWIL1 did not affect the mRNA levels of *CLOCK* and *BMAL1* in PIWIL1 overexpression or knockdown HeLa and MCF7 cells (C). PIWIL1 positively regulated CLOCK and BMAL1 expression both in cytoplasm and nucleus, GAPDH and LaminB1 were employed as internal controls (D). Data were presented as mean ± SD (N = 3). NS, no significant change. *P < 0.05

First, we screened cancer cell lines for PIWIL1 expression, and the results showed that PIWIL1 was expressed in HeLa, MCF7, HepG2, A549 and HT‐29 cancer cells (Figure S1A,B). To investigate the effect of PIWIL1 on circadian proteins of CLOCK and BMAL1, we constructed over‐expression and shRNA expression vectors of PIWIL1, which were injected into Hela and MCF7 cells. The Western blot results showed that up‐regulated PIWIL1 can increase CLOCK and BMAL1 expression, while PIWIL1 knockdown decreased CLOCK and BMAL1 expression (Figure 1A and Figure S2). Subsequently, immunofluorescence assays was performed and indicated that PIWIL1 increased CLOCK and BMAL1 expression in Hela and MCF7 cells (Figure 1B).

The changes of CLOCK and BMAL1 protein levels may be due to proteins degradation or changes at mRNA levels. We first performed the real‐time PCR and no significant changes were observed after altering the PIWIL1 expression (Figure 1C). Interesting, the cytosolic/nuclear fractionation assays further revealed that PIWIL1 positively regulated CLOCK and BMAL1 expression both in cytoplasm and nucleus (Figure 1D). Above results suggested that PIWIL1 plays a role in regulating circadian proteins.

### 3.2 PIWIL1 interacts with CLOCK and BMAL1 via PIWI domain

**Figure 2:**
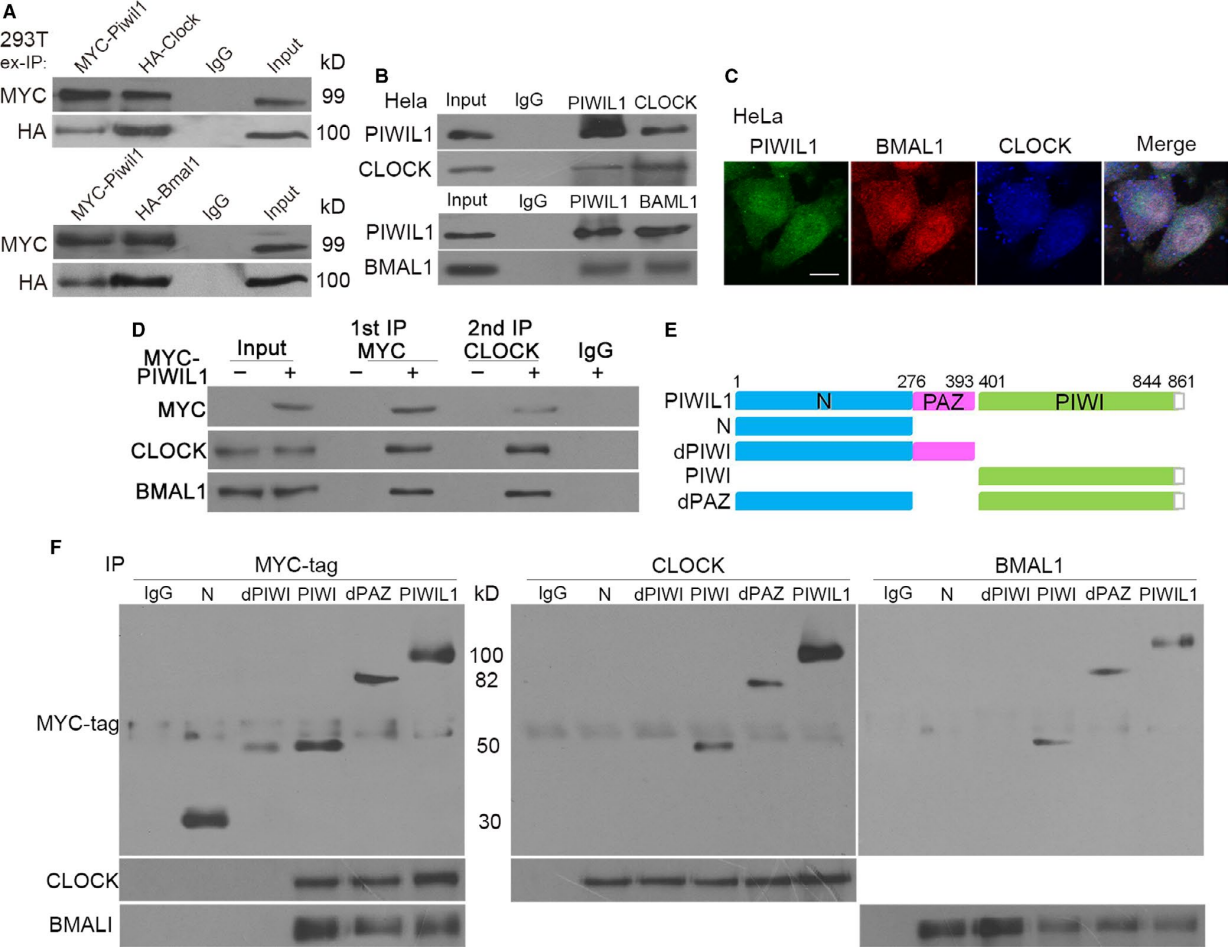
PIWIL1 interacts with CLOCK and BMAL1 via PIWI domain. Exogenous Immunoprecipitation (IP) assays showed that PIWIL1 interacted with CLOCK and BMAL1 in 293T. Input, protein sample without immunoprecipitation (A). Endogenous IP results revealed that PIWIL1 bound with CLOCK and BMAL1 in Hela (B). Immunofluorescence assays showed that PIWIL1, CLOCK and BMAL1 were overlapped in cytoplasm and nucleus. Scale bar corresponds to 10 μm (C). Two‐step IP results indicated that PIWIL1, CLOCK and BMAL1 formed complex (D). Schematic of PIWIL1 deletion mutants (E). PIWIL1 deletion mutants contained PIWI domain can interact with CLOCK and BAML1 in Hela cells (F)

Evidences have shown that PIWIL1 can regulate CLOCK and BMAL1, so we next confirmed whether PIWIL1 can interact with CLOCK and BMAL1. IP assays were performed, showing that exogenous and endogenous PIWIL1 can interact with CLOCK and BMAL1 in 293T and Hela cells (Figure 2A,B). Then immunofluorescence assays showed that PIWIL1, CLOCK and BMAL1 were overlapped in cytoplasm and nucleus (Figure 2C), suggesting the existence of complex. Subsequently, two‐step immunoprecipitation assays further confirmed the complex comprised of PIWIL1, CLOCK and BMAL1 (Figure 2D).

To identify the functional domains involved in interaction with CLOCK and BMAL1, we constructed a series of PIWIL1 deletion mutants (Figure 2E). Immunoprecipitation assays showed that the mutants of PIWIL1 lacking of PIWI domain such as N or dPIWI failed to interact with CLOCK and BMAL1, only mutants contained PIWI domain remained the ability in Hela cells (Figure 2F), verifying the importance of PIWI domain.

### 3.3 PIWIL1 suppresses GSK3β‐induced phosphorylation and ubiquitination of CLOCK and BMAL1

**Figure 3:**
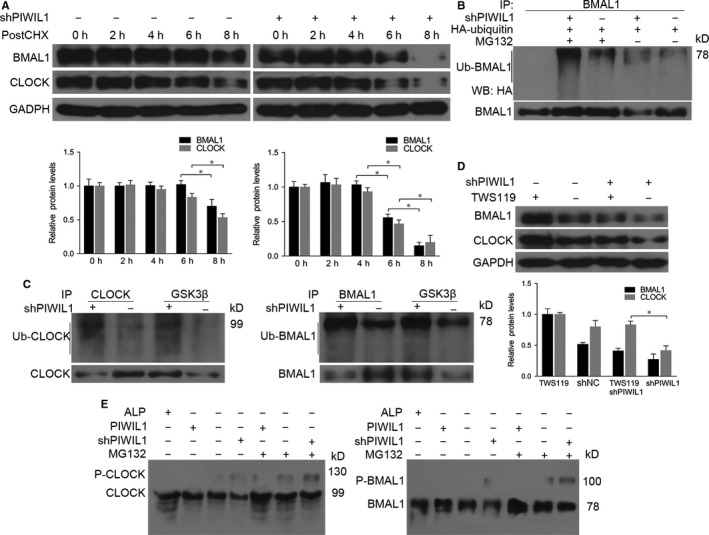
PIWIL1 suppresses GSK3β‐induced phosphorylation and ubiquitination of CLOCK and BMAL1. PIWIL1 knockdown increased degradation of CLOCK and BMAL1 compared with Control after Hela cells were treated with cycloheximide (CHX) at 6 μg/mL for indicated time (hours) (A). Ubiquitination assay showed that PIWIL1 knockdown increased the level of poly‐ubiquitination of BMAL1. HeLa cells were transfected with HA‐ubiquitin vector, followed with treatment of MG132 for 6 h (B). Anti‐GSK3β can pull down more CLOCK, BMAL1 and ubiquitinated CLOCK and BMAL1 in PIWIL1 knockdown cells (C). GSK3β inhibitor TWS119 recovered CLOCK and BMAL1 expression decreased by PIWIL1 knockdown Hela cells (D). Phos‐tag SDS PAGE assays indicated that PIWIL1 inhibited the phosphorylation of CLOCK and BMAL1. ALP, alkaline phosphatase (E). Data were presented as mean ± SD (N = 3). *P < 0.05

As we all know that phosphorylation and ubiquitination of CLOCK and BMAL1 significantly contribute to the stability of these proteins, and can regulate circadian rhythms.^10^ In Hela cells treated with CHX to inhibit protein synthesis, PIWIL1 knockdown increased degradation of CLOCK and BMAL1 (Figure 3A). Ubiquitination assays showed that PIWIL1 knockdown increased the levels of poly‐ubiquitination of BMAL1 (Figure 3B).

Previous studies showed that GSK3β can induce phosphorylation and ubiquitination of CLOCK and BMAL,^10^, ^19^, ^23^ so we would like to understand whether PIWIL1 can block GSK3β‐induced phosphorylation and degradation of CLOCK and BMAL1. Immunoprecipitation assays showed that anti‐GSK3β can pull down more CLOCK, BMAL1 and ubiquitinated CLOCK and BMAL1 in PIWIL1 knockdown cells (Figure 3C). Next we treated the cells with GSK3β inhibitor TWS119 and harvested for Western blot, the results revealed that GSK3β inhibitor TWS119 recovered CLOCK and BMAL1 expression decreased by PIWIL1 knockdown (Figure 3D).

To further look into the impact of PIWIL1 on phosphorylation of CLOCK and BMAL1, Phos‐tag SDS PAGE gels assays were performed to separate phosphorylated proteins and the results showed that PIWIL1 can inhibit the phosphorylation of CLOCK and BMAL1 (Figure 3E).

### 3.4 PIWIL1 activates SRC‐PI3K‐AKT pathway to suppress GSK3β activity

**Figure 4:**
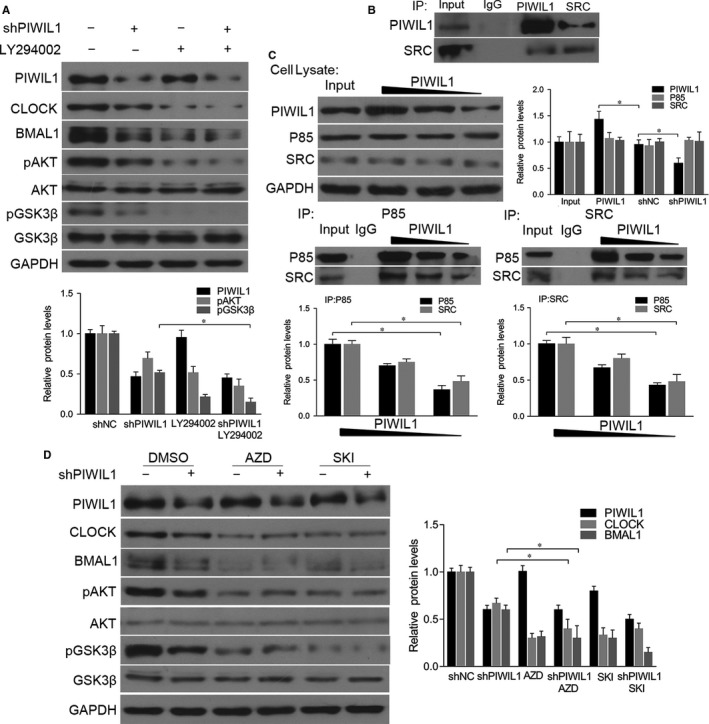
PIWIL1 activates SRC‐PI3K‐AKT pathway to repress GSK3β activity. PIWIL1 knockdown decreased the level of phosphorylation of GSK3β in Hela. This effect can be abolished by LY294002, an inhibitor of PI3K (A). Immunoprecipitation (IP) results showed that PIWIL1 interacted with SRC in Hela cells (B). PIWIL1 positively regulated the interaction between SRC and p85 regulatory subunit of PI3K (C). SRC inhibitors (AZD, SKI) attenuated PIWIL1‐dependent stability of CLOCK and BMAL1 (D). Data were presented as mean ± SD (N = 3). *P < 0.05

As a kinase, GSK3β is effective in unstimulated cells and can be regulated by phosphorylation at Ser9 by other kinase,^24^, ^25^ so we examined whether PIWIL1 can suppress the GSK3β activity. The results showed that PIWIL1 knockdown significantly decreased the level of phosphorylation of GSK3β at Ser9 in Hela cell. And this effect can be abolished by LY294002, an inhibitor of PI3K (Figure 4A), showing that PIWIL1 inhibited GSK3β activity via PI3K pathway.

Studies have shown that SRC is involved in PI3K‐induced AKT phosphorylation to regulate PI3K‐AKT pathway.^26^, ^27^, ^28^ So we hypothesized that PIWIL1 can activate PI3K‐AKT pathway via SRC to phosphorylate AKT and GSK3β. First, the immunoprecipitation assays showed that PIWIL1 can interact with SRC (Figure 4B). Then we observed that PIWIL1 overexpression increased the interaction between SRC and p85 regulatory subunit of PI3K, while PIWIL1 knockdown can weaken the interaction (Figure 4C). Furthermore, when inhibitors of SRC, AZD and SKI were introduced, CLOCK and BMAL1 expression were significantly decreased, as well as phosphorylation level of GSK3β, and PIWIL1 knockdown cannot restore this effect of SRC inhibitors (Figure 4D). Taken together, PIWIL1 promotes the stability of CLOCK and BMAL1 proteins in a SRC‐dependent manner.

### 3.5 PIWIL1 suppresses transcriptional activation activity of E‐BOX

**Figure 5:**
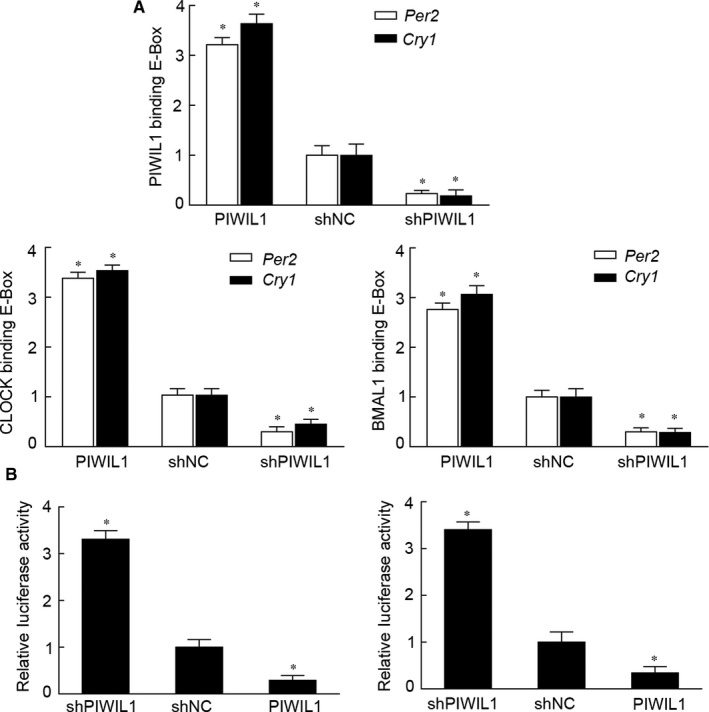
PIWIL1 suppresses transcriptional activity of E‐BOX. Chromatin immunoprecipitation results showed that PIWIL1 bound with E‐BOX regions on promoters of *PER2* and *CRY1*, and enhanced this interaction between CLOCK/BMAL1 and E‐BOX region (A). Dual luciferase reporter gene assays revealed that PIWIL1 overexpression can suppress transcriptional activities of E‐BOX on promoters of *PER2* and *CRY1*; and *vice versa*. Data were presented as mean ± SD (N = 3). *P < 0.05 (B)

We have provided evidences that PIWIL1 can enhance the stability of CLOCK and BMAL1, we would like to know whether PIWIL1 can regulate CCG by recruiting CLOCK/BMAL1 complex to CCG promoters on E‐BOX region. ChIP was performed to explore whether PIWIL1 can enhance the interaction between CLOCK/BMAL1 and E‐BOX region, and our results showed that PIWIL1 significantly enhanced this interaction. Interestingly, we first showed that PIWIL1 can bind with E‐BOX regions on promoters of *PER2* and *CRY1*, suggesting its novel function in gene transcription (Figure 5A and Figure S3A).

Next, reporter gene vectors with E‐BOX elements of *PER2* and *CRY1* were constructed. Dual luciferase reporter gene assays revealed that PIWIL1 overexpression can suppress transcriptional activation activities on promoters of *PER2* and *CRY1*; and *vice versa* (Figure 5B and Figure S3B). Taken together, PIWIL1 can negatively regulate transcriptional activities of E‐BOX region on promoters of *PER2* and *CRY1*.

### 3.6 PIWIL1 knockdown recovers circadian rhythms in cancer cells

**Figure 6:**
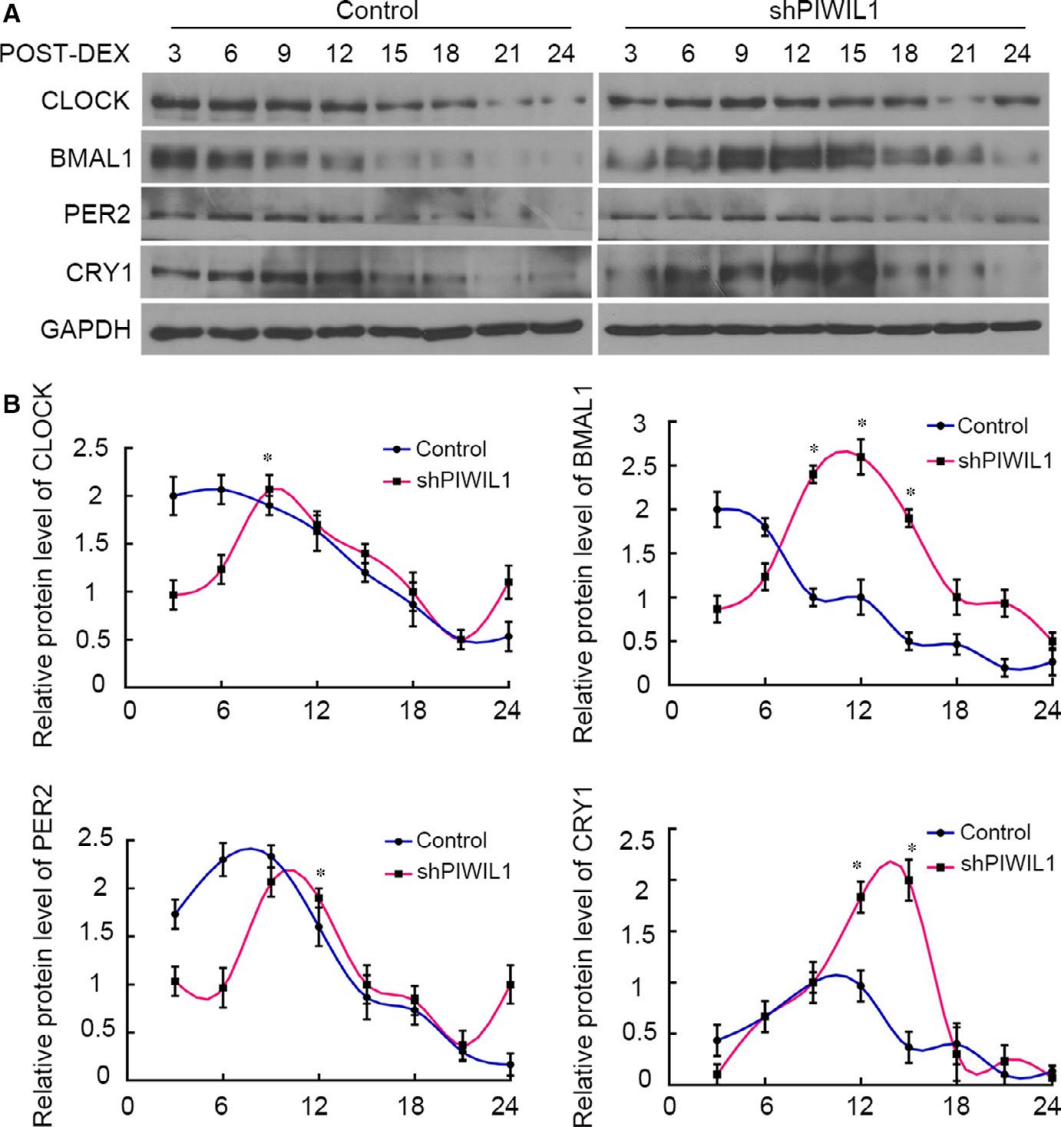
PIWIL1 knockdown recovers circadian rhythms in cancer cells. CLOCK, BMAL1, PER2 and CRY1 exhibited approximately 24 hours’ circadian rhythms in PIWIL1 knockdown cells (A). The intensity of bands was quantified by Quantity One software, the data were normalized and represented as mean ± SD (N = 3). *P < 0.05 (B)

Above results have proved that PIWIL1 can regulate CLOCK and BMAL1, we thereby explored whether PIWIL1 impacted on circadian rhythms. Control (WT HeLa cells) and shPIWIL1 Hela cells were first synchronized by dexamethesone and harvested for Western blot. The results showed that the expressions of CLOCK, BMAL1, PER2 and CRY1 did not exhibit obvious circadian rhythms in Control (WT HeLa cells) group. However, when PIWIL1 was knockdown, circadian proteins exhibited approximately 24 hours’ circadian rhythms (Figure 6A,B). Taken together, PIWIL1 knockdown can recover circadian rhythms of circadian genes to a certain extent by regulating CLOCK and BMAL1 post‐translation modifications.

## 4 DISCUSSION

Recent researches have shown that PIWIL1 is involved in tumorigenesis,^29^, ^30^, ^31^ meanwhile, the tumorigenesis is associated with circadian rhythms in humans.^21^, ^22^ Then our immunoprecipitation results showed that PIWIL1 can interact with circadian core proteins CLOCK and BMAL1 in cancer cells, suggesting the linkage between PIWIL1 and circadian rhythms. Subsequently, we have found that PIWIL1 can be as the upstream regulator of circadian network to positively regulate clock proteins, showing that PIWIL1 can suppress circadian rhythms.

The circadian rhythms are maintained by self‐sustained transcriptional and post‐translational feedback loops,^1^, ^32^, ^33^ and the PTMS of clock proteins contribute to regulate circadian rhythms.^34^, ^35^, ^36^, ^37^ Our results first showed PIWIL1 can promote the stability of CLOCK and BMAL1 through GSK3β‐induced phosphorylation and ubiquitination degradation of CLOCK and BMAL1 (Figure 3). Then we gave the evidences that PIWIL1 promotes SRC interacting with PI3K, activates PI3K‐AKT signalling pathway, phosphorylates and inactivates GSK3β. Thus, with kinase activity loss of GSK3β, CLOCK and BMAL1 cannot be appropriately phosphorylated, ubiquitinated and degraded (Figure 4).

Furthermore, the impact of PIWIL1 on circadian rhythms is not limited to suppress the kinase activity of GSK3β but also can bind to E‐BOX region of CCG promoter and enhance the interaction between CLOCK/BMAL1 and E‐BOX region. Interestingly, by performing dual luciferase report gene assays, the results revealed that PIWIL1 can negatively regulate transcriptional activation activities of E‐BOX on promoters of *PER2* and *CRY1* in Hela cells (Figure 5), extending the nuclear function of PIWIL1.

Above results revealed that PIWIL1 can lead to accumulation of the CLOCK and BMAL1 proteins and then disrupt the transcription‐translation feedback loops. Next, our further discoveries showed that PIWIL1 knockdown can recover the circadian rhythms to a certain extent in HeLa cancer cells (Figure 6), first indicating that PIWIL1 can play as a regulator for circadian clock. Circadian clock controls about 5%–10% genes expressions in mammal genome, involving in various physiological processes,^38^, ^39^ therefore, the effects of PIWIL1 may extend to more CCG and signalling pathways.

**Figure 7:**
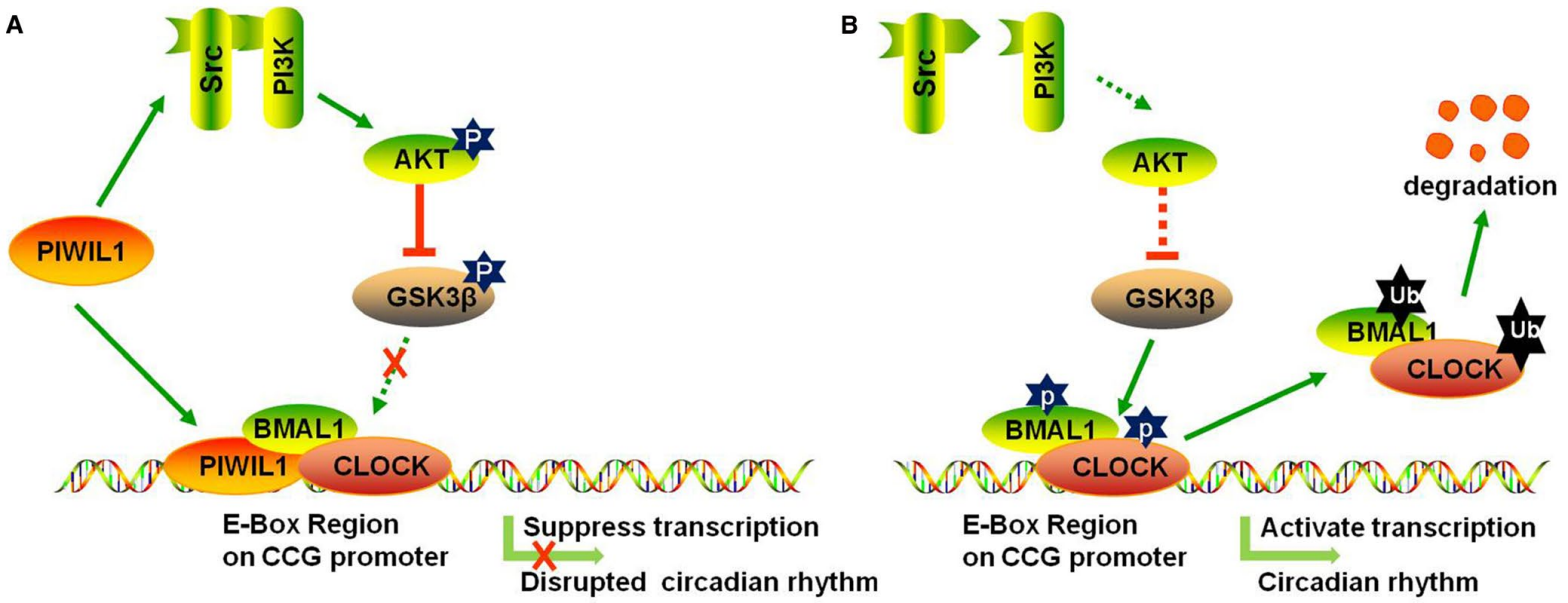
Schematic model for PIWIL1 regulating circadian rhythms

Collectively, our work provided the evidences showing that PIWIL1 regulates circadian rhythms at least via two pathways in cancer cells: (a) To activate SRC‐PI3K‐AKT signalling pathway to phosphorylate and inactivate GSK3β, preventing CLOCK and BMAL1 from GSK3β‐induced phosphorylation and degradation; (b) To bind with E‐BOX region together with CLOCK/BMAL1 complex to repress the transcriptional activities of *PER2* and *CRY1* promoters (Figure 7). Taken together, our findings reveal a novel function of PIWIL1 in regulating circadian rhythms, providing molecular connection between dysfunction of circadian rhythms and tumorigenesis.

## CONFLICT OF INTEREST

The authors declare no conflicts of interest.

## AUTHORS’ CONTRIBUTIONS

Hao Tan and Yongxin Ma designed the research; Hao Tan performed, analysed the experiments and data; Yilu Lu and Yingchuan Zhu reviewed the article; Dachang Tao and Yunqiang Liu contributed the reagents and analytic tools; Hao Tan and Yongxin Ma wrote the manuscript.

## Data Availability

The data used to support the findings of this study are available from the corresponding author upon request.
