# Supplementary figures and images for: PIWIL1 suppresses circadian rhythms through GSK3β‐induced phosphorylation and degradation of CLOCK and BMAL1 in cancer cells
Source: J Cell Mol Med. 2019 May 16;23(7):4689–98. doi: 10.1111/jcmm.14377 (PMC6584488; doi:10.1111/jcmm.14377)

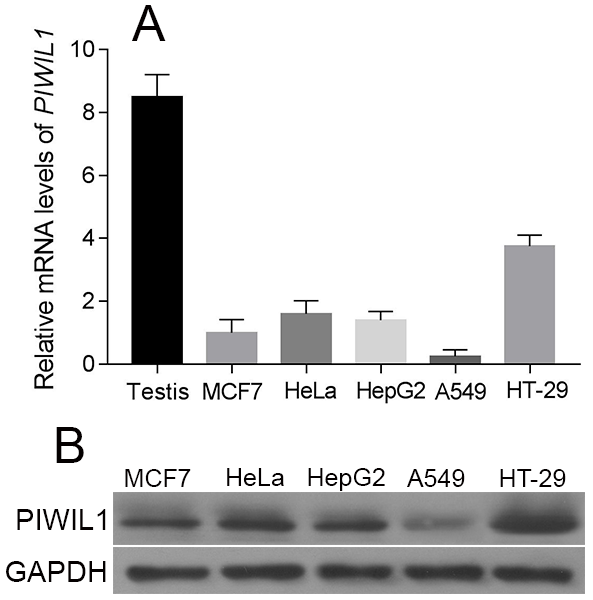

Supplement: Supplementary file 1 [file JCMM-23-4689-s001.tif]

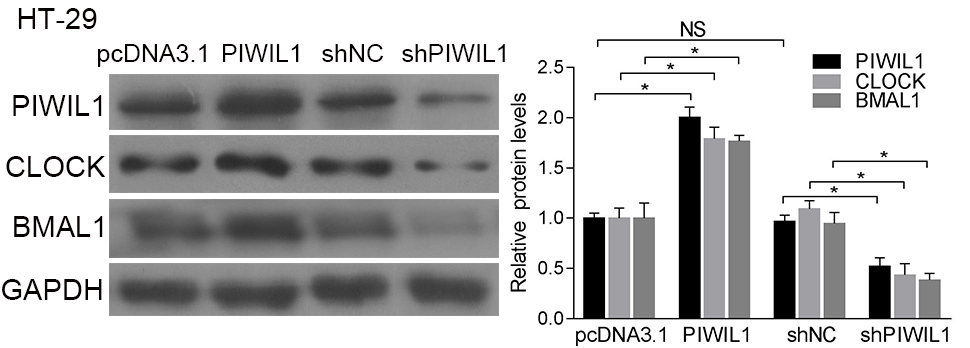

Supplement: Supplementary file 2 [file JCMM-23-4689-s002.tif]

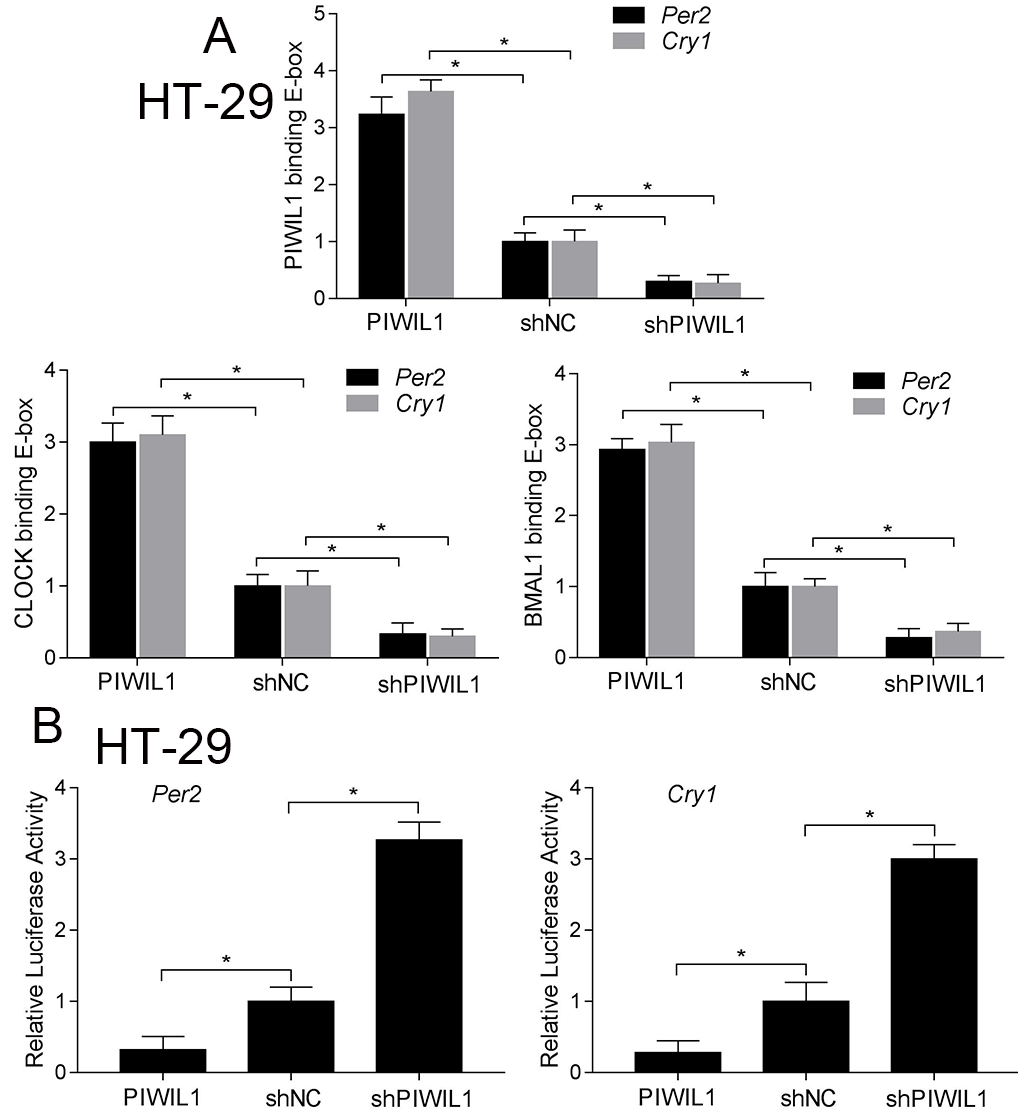

Supplement: Supplementary file 3 [file JCMM-23-4689-s003.tif]
